# 培美曲塞及吉西他滨分别联合顺铂治疗初治晚期非小细胞肺癌安全性和有效性的随机对照研究

**DOI:** 10.3779/j.issn.1009-3419.2012.10.02

**Published:** 2012-10-20

**Authors:** 兴胜 胡, 顺昌 焦, 树才 张, 哲海 王, 孟昭 王, 诚 黄, 荣生 郑, 凯 李, 洁 王, 雅杰 王, 学农 欧阳, 文光 吕, 刚 程, 春宏 胡, 荣城 罗, 燕 孙

**Affiliations:** 1 100021 北京，中国医学科学院肿瘤医院 Cancer Institute & Hospital, Chinese Academy of Medical Sciences, Beijing 100021, China; 2 100853 北京，中国人民解放军总医院 Chinese PLA General Hospital, Beijing 100853, China; 3 100095 北京，首都医科大学附属北京胸科医院 Beijing Chest Hospital, Capital Medical University, Beijing 100095, China; 4 250117 济南，山东省肿瘤医院 Shandong Provincial Cancer Hospital, Jinan 250117, China; 5 100730 北京，中国医学科学院北京协和医院 Chinese Academy of Medical Sciences & Peking Union Medical College, Beijing 100730, China; 6 350014 福州，福建省肿瘤医院 Fujian Provincial Cancer Hospital, Fuzhou 350014, China; 7 233000 蚌埠，蚌埠医学院第一附属医院 First Affiliated Hospital of Bengbu Medical College, Bengbu 233000, China; 8 300060 天津，天津市肿瘤医院 Tianjin Cancer Hospital, Tianjin 300060, China; 9 100142 北京，北京肿瘤医院 Beijing Cancer Hospital, Beijing 100142, China; 10 200433 上海，第二军医大学附属长海医院 Changhai Hospital, Shanghai 200433, China; 11 350025 福州，南京军区福州总医院 Fuzhou General Hospital of Nanjing Military Command, Fuzhou 350025, China; 12 300191 天津，天津市人民医院 Nankai University Affiliated Hospital, Tianjin 300191, China; 13 100730 北京，卫生部北京医院 Beijing Hospital, Beijing 100730, China; 14 410001 长沙，中南大学湘雅二医院 The Second Xiangya Hospital of Central South University, Changsha 410001, China; 15 510515 广州，南方医科大学南方医院 Nanfang Hospital, Guangzhou 510515, China

**Keywords:** 非小细胞肺癌, 化疗, 培美曲塞, 吉西他滨, 顺铂, NSCLC, Chemotherapy, Pemetrexed, Gemcitabine, Cisplatin

## Abstract

**背景与目的:**

由于不同个体间肿瘤细胞的生物学特性差异以及现有药物在疗效和不良反应方面仍存在的一定缺陷，故本研究旨在比较培美曲塞及吉西他滨分别联合顺铂治疗初治晚期非小细胞肺癌（non-small cell lung cancer, NSCLC）的临床疗效及毒副作用。

**方法:**

251例患者被随机分为培美曲塞联合顺铂组（PP组）127例和吉西他滨联合顺铂组（GP组）124例。PP组：培美曲塞500 mg/m^2^，d1，顺铂75 mg/m^2^，d1。GP组：吉西他滨1, 000 mg/m^2^，d1, 8，顺铂75 mg/m^2^，d1。两组治疗周期均为每3周1次。此外，两组均合并给药叶酸、VitB_12_和地塞米松。

**结果:**

PP组和GP组患者的总有效率分别为25.20%和17.74%，其中非鳞癌患者总有效率分别为27.62%和16.00%。两组肿瘤进展时间分别为6.5个月和5.6个月，中位生存期分别为16.9个月和17.0个月，1年生存率分别为59.62%和65.87%，2年生存率分别为27.28%和27.93%。PP组非鳞癌的总有效率明显高于GP组，结果具有统计学意义。但两组总缓解率、肿瘤进展时间、中位生存期、1年及2年生存率均无明显性差异。从不良反应来看，PP组的白细胞减少、血小板降低、血红蛋白降低、脱发等不良反应明显低于GP组，结果具有统计学意义。

**结论:**

培美曲塞联合顺铂治疗晚期非小细胞肺癌与吉西他滨联合顺铂疗效相当，但副作用明显减少。总之，培美曲塞联用顺铂可以作为安全有效的药物对初治的非小细胞肺癌进行临床一线治疗。

## Abstract (en)

**Background and objective:**

Due to the various inter-individual differences in the biological characteristics of tumor cells, as well as issues on the efficacy, adverse reactions, and defects of existing drugs, we compared the clinical efficacy and toxicity of pemetrexed and gemcitabine combined with cisplatin for the treatment of previously untreated advanced non-small cell lung cancer (NSCLC).

**Methods:**

251 patients were randomly divided into pemetrexed combined with cisplatin group (PP group) with 127 cases and gemcitabine combined with cisplatin group (GP group) with 124 cases. PP group received pemetrexed 500 mg/m^2^ iv infusion d1 and cisplatin 75 mg/m^2^ iv infusion d1, whereas GP group received gemcitabine 1, 000 mg/m^2^ iv infusion d1, 8 and cisplatin 75 mg/m^2^ iv infusion d1. The treatment cycle was once every three weeks. In addition, folic acid, vitamin B_12_, and dexamethasone were administered in both groups.

**Results:**

The total clinical effective rates in PP group and GP group were 25.20% and 17.74%, respectively. The total efficiencies of non-squamous cell carcinoma were 27.62% and 16.00%. The tumor progression duration in these two groups was 6.5 and 5.6 months, respectively. The median survival time in the two groups was 16.9 and 17.0 months, respectively, with 59.62% and 65.87% survival rates of 1 year and 27.28% and 27.93% survival rates of 2 years, respectively. The total efficacy of non-squamous cell carcinoma in the PP group was significantly higher than that in GP group. The results were statistically significant. However, there were no significant differences in total response rates, tumor progression duration, and median survival rates of 1 and 2 years. The rate of adverse reactions, including white blood cell reduction, lower platelet count, lower hemoglobin, and hair loss in the PP group was significantly lower than that in the GP group. The results were statistically significant.

**Conclusion:**

The clinical efficacy of pemetrexed and gemcitabine combined with cisplatin for the treatment of previously untreated advanced NSCLC was roughly the same, but the adverse reactions decreased significantly in the PP group compared with those in the GP group. Therefore, pemetrexed combined with cisplatin can be used as a safe and effective drug for clinical first-line treatment for previously untreated NSCLC.

目前，肺癌已经成为全球范围内发病率及死亡率最高的恶性肿瘤之一，其发病率仍呈持续上升趋势。其中约80%的肺癌为非小细胞肺癌（non-small cell lung cancer, NSCLC）^[1]^。由于早期诊断技术的局限性及缺乏早期特异性临床表现，约70%-80%的患者确诊时已属晚期。因此如何找到安全、有效的药物治疗肺癌已成为肺癌防治工作者面临的严肃而紧迫的任务。吉西他滨是嘧啶类抗代谢药物，是目前NSCLC的常用化疗药物之一。然而由于不同个体间肿瘤细胞的生物学特性差异较大，对化疗药物的敏感性差异亦大，使其在总有效率及不良反应方面具有一定的局限性。培美曲塞（pemetrexed）为一种多靶点抗叶酸制剂，具有抑制肿瘤细胞增殖的目的^[2]^。因其有效性和良好的耐受性，在2008年9月被美国食品药品管理局（Food and Drug Administration, FDA）批准培美曲塞与顺铂联用作为治疗晚期非小细胞肺癌的一线治疗方法^[3]^。

为进一步探讨培美曲塞联合顺铂是否可以作为我国治疗晚期NSCLC的安全有效的方案推向临床一线使用，我院联合14家单位在2008年3月-2009年10月采用培美曲塞和吉西他滨分别与顺铂联合治疗局部晚期或转移性NSCLC初治患者，对比两种方案的疗效及不良反应，以期为临床应用提供参考。

## 1 资料与方法

### 1.1 一般资料

251例年龄为18岁-70岁，性别不限的局部晚期或转移性NSCLC初治患者被随机分为PP组127例和GP组124例。入选标准为：①经病理学/细胞学确诊的初治局部晚期和转移性（Ⅲb期或Ⅳ期）的不能进行根治手术的NSCLC患者，或既往手术，术后辅助化疗结束6个月后复发，并且未经培美曲塞、吉西他滨治疗者；②至少有一个客观可测量（影像学：CT或MRI）的肿瘤病灶，采用常规测量技术测量最大径≥20 mm，或采用螺旋CT和MRI扫描最大径≥10 mm；③ECOG评分≤1分，预计生存时间≥3个月；④WBC≥4.0×10^9^/L，ANC≥1.5×10^9^/L，Hb≥90 g/L，PLT≥100×10^9^/L，血清胆红素≤1.5×常值高限，AST、ALT≤1.5×正常值高限，BUN、Cr≤正常值上限，肌酐清除率≥45 mL/min，心电图基本正常；⑤入选患者为没有症状的脑转移患者；且依从性好并签署知情同意书。治疗前两组性别、年龄、临床分期、ECOG评分均无明显差异（*P* > 0.05）（表 1）。

**1:** 两组患者一般资料比较 The characteristics of the two groups

Characteristics		PP group (*n*=127)	Gp group (*n*=124)	*P*
Gender	Male	78 (61.42%)	77 (62.10%)	0.911, 8
	Female	49 (38.58%)	47 (37.90%)	
Median age/yr		54	55	0.136, 9
Stage	Ⅲ	45 (35.43%)	35 (28.23%)	0.220, 5
	Ⅳ	82 (64.57%)	89 (71.77%)	
ECOG scores	0	43 (33.86%)	44 (35.48%)	0.786, 7
	1	84 (66.14%)	80 (64.52%)	
Pathology	Adenocarcinoma	94 (74.02%)	88 (70.97%)	0.470, 8
	Squmocellular carcinana	22 (17.32%)	24 (19.35%)	
	Large-cell carcinoma	5 (3.94%)	2 (1.61%)	
	Others	6 (4.72%)	10 (8.06%)	

### 1.2 治疗方法

培美曲塞联合顺铂组（PP组）127例：培美曲塞（齐鲁制药：商品名赛珍）500 mg/m^2^，d1，顺铂75 mg/m^2^，d1，每3周1次。吉西他滨联合顺铂组（GP组）124例：吉西他滨（豪森制药：商品名泽菲）1, 000 mg/m^2^，d1, 8，顺铂75 mg/m^2^，d1。两组保持用药平衡均合并给药叶酸、VitB_12_和地塞米松。

### 1.3 疗效及毒性反应评定标准

治疗2个周期后，使用RECIST标准评价疗效，如果1次化疗后患者主诉症状加重，也需要评价疗效，并在首次评价完全缓解（complete response, CR）、部分缓解（partial response, PR）、稳定（stable disease, SD）之后4周加以复核确认。按NCI-CTC 3.0常见毒性分级标准评价毒副反应。每周复查2次血常规、每周期复查肝肾功能、心电图。采用QOL测定量表FACT-L（4.0）中文版，由统一培训的医学专业人员对可随访到的肺癌患者进行直接访问方式问卷调查。

### 1.4 统计分析

采用SPSS 16.0软件进行统计分析，卡方检验、*Fisher’s*精确检验及秩和检验比较组间临床特征、疗效及毒副反应的差异；应用*Kaplan-Meier*单因素方法进行生存分析，*Log-rank*检验判断生存情况差异。*P* < 0.05为差异具有统计学意义。

## 2 结果

治疗期间共脱落39例。其中PP组18例，包括病情进展1例，毒性反应2例，患者中止治疗7例，死亡3例，失访4例，严重违背方案1例；GP组21例，包括毒性反应6例，患者中止治疗7例，死亡1例，严重违背方案规定2例，失访3例，出现脑部症状1例，继续化疗患者不能受益1例。

### 2.1 两组疗效比较

两组最多均完成6周期化疗，均无CR病例。PP组可评价疗效为127例，中位化疗周期数为5周期。PR 32例（25.20%），SD 55例（43.30%），PD 40例（31.50%）。其中非鳞癌患者可评价疗效105例，PR 29例（27.62%），SD 47例（44.76%），PD 29例（27.62%）。腺癌患者可评价疗效94例。PR 28例（29.79%），SD 43例（45.74%），PD 23例（24.47%）。GP组可评价疗效为124例，中位化疗周期数为4周期。PR 22例（17.74%），SD 48例（38.76%），PD 54例（43.50%）。其中非鳞癌患者可评价疗效100例，PR 16例（16.00%），SD 68例（68.00%），PD 16例（16.00%）。腺癌患者可评价88例，PR 15例（17.05%），SD 35例（39.77%），PD 38例（43.18%）。结果发现，两组间总缓解率统计学无差异；但亚组分析中，PP组中非鳞癌患者及腺癌患者的总缓解率高于GP组，结果具有统计学差异（表 2-表 4）。

**2:** 两组总缓解率的情况及比较 Comparison of difference of total remission rate

Item	PP group (*n*=127)	GP group (*n*=124)
PR	32	22
Total remission rate (%)	25.20	17.74
Difference of total remission rate (%)	7.46	
*P*	0.124, 2	

**3:** 非鳞癌的患者总缓解率比较 Comparison of difference of total remission rate of no squmocellular carcinona

Item	PP group (*n*=105)	GP group (*n*=100)
PR	29	16
Total remission rate (%)	27.62	16.00
Difference of total remission rate (%)	11.62	
*P*	0.031, 8	

**4:** 腺癌的患者总缓解率比较 Comparison of difference of total remission rate of adenocarcinoma

Item	PP group (*n*=94)	GP group (*n*=88)
PR	28	15
Total remission rate (%)	29.79	17.05
Difference of total remission rate (%)	12.74	
*P*	0.035, 8	

### 2.2 生存情况

本研究共计251例患者，随访至2011年12月，中位随访时间18个月，仍然生存患者共计22例（8.66%），死亡患者191例（75.20%），失访患者38例（16.14%）。PP组及GP组全部患者肿瘤进展时间分别为6.5个月和5.6个月，其中非鳞癌患者分别为6.6个月和5.1个月。两组之间差异无统计学意义（*P*=0.610, 5）。两组中位生存时间分别为16.9个月和17.0月，（按照每个月30天计算），其中非鳞癌患者分别为17.4个月和17.0个月，亦无统计学差异（*P*=0.520, 5）。此外还对两组患者1年及2年生存率进行比较，亦无统计学差异。两组生存情况见表 5。

**5:** 生存情况比较 Comparison of survival between two groups

Item	PP group (*n*=127)	GP group (*n*=124）	*P*
Time to progress (month)	6.5	5.6	0.610, 5
Median survival time (month)	16.9	17.0	0.520, 5
1-year survival rate (%)	59.62%	65.87%	0.132, 8
2-year survival rate (%)	27.28%	27.93%	0.150, 2

### 2.3 不良反应和生活质量评价

两组患者均出现血液学毒性以及恶心、呕吐、便秘、脱发、肝肾功能损害等不良反应，PP组患者无论血液学还是非血液学毒性，其发生率均明显低于GP组。其中白细胞减少、血小板降低、血红蛋白降低、脱发等不良反应结果具有统计学意义。见表 6。通过QOL评分进行综合分析，PP组的QOL得分为129±13，GP组得分为127±19，其结果无统计学差异（*P* > 0.05）。

**6:** 各种不良反应发生情况及比较 Comparison of toxity between two groups

Side-effects	PP group				GP group			*P*
	Total examples	Occurrence examples	Incidence rate (%)		Total examples	Occurrence examples	Incidence rate (%)	
Granulocytopenia	251	88	68.75		252	94	74.60	0.300, 7
Nauseated	213	78	60.94		195	68	53.97	0.261, 3
Leukopenia	216	75	58.59**		279	94	74.60	0.006, 9
Lower hemoglobin	157	64	50.00**		227	84	66.67	0.007, 1
Vomiting	118	54	42.19		111	54	42.86	0.914, 0
Lower platelet	45	22	17.19***		114	59	46.83	< 0.000, 1
Constipation	45	21	16.41		52	29	23.02	0.185, 3
Poor appetite	47	13	10.16		56	19	15.08	0.237, 1
Higher propylene ammonia transaminase	17	10	7.81		16	11	8.73	0.790, 6
Acratia	14	8	6.25		20	14	11.11	0.168, 5
Hypocalcemia	12	8	6.25		7	6	4.76	0.603, 3
Baldness	15	7	5.47*		38	19	15.08	0.011, 5
Diarrhea	8	7	5.47		8	7	5.56	0.975, 8
Renal function abnormal	9	6	4.69		9	6	4.76	0.977, 7
Lower albumin	6	5	3.91		8	4	3.17	0.999, 9
Stomatitis	6	5	3.91		6	3	2.38	0.736, 4
Comparison with GP group, ^*^P < 0.05, ^**^P < 0.01, ^***^P < 0.001.								

## 3 讨论

自20世纪90年代以来，第三代化疗药物吉西他滨与顺铂联合方案已成为晚期NSCLC的标准一线治疗方案^[4]^。研究表明，吉西他滨虽对多种实体瘤如NSCLC疗效较好，但不容忽视的是此方案易导致白细胞和血小板减少从而引发骨髓毒性症状。因而一定程度上影响了临床的推广使用^[5, 6]^。随着新型多靶点抗叶酸药物的出现，为已陷入瓶颈的NSCLC的临床药物治疗带来新的希望。但培美曲塞联合铂类方案是否具有更好的疗效和安全性，需要大样本、多中心的临床试验来证实。

培美曲塞（PEM）是一种新型的多靶点抗叶酸药物，作用于叶酸依赖性代谢途径中的多个酶，包括胸苷酸合成酶（TS）、二氢叶酸还原酶（DHFR）和甘氨酰胺核苷甲酰基转移酶（GARFT）等。通过多靶点抑制这些关键酶活性，导致嘌呤和嘧啶合成障碍，使得肿瘤细胞的增殖停滞于S期从而控制肿瘤细胞的生长^[7]^。培美曲塞与铂类药物联合一线治疗晚期NSCLC的Ⅱ期临床研究^[8, 9]^表明，培美曲塞/铂类联合方案的疗效与其他常用的含铂两药方案相似，而毒性反应的发生率则明显较低。国外临床试验^[10, 11]^观察了培美曲塞联合顺铂对比吉西他宾联合顺铂一线治疗NSCLC的疗效和不良反应。结果显示两组疗效无明显差异，培美曲塞联合顺铂组与吉西他宾联合顺铂组的OS均为10.3个月，PFS分别为4.8个月和5.1个月，1年生存率分别为43.5%和41.9%，ORR分别为30.6%和28.2%。在不良反应方面两组的耐受性均较好，但除恶心外培美曲塞联合顺铂组不良反应发生率明显低于吉西他滨联合顺铂组。亚组分析显示，培美曲塞联合顺铂组与吉西他滨联合顺铂组相比在腺癌和大细胞癌中OS明显延长，腺癌分别为12.6个月和10.9个月，大细胞癌分别为10.4个月和6.7个月。腺癌和大细胞癌OS有统计学差异。而在鳞状细胞癌中两组的OS分别为9.4个月和10.8个月，没有统计学差异。

本研究中PP组和GP组全部患者FAS集1年生存率分别为59.62%和65.87%，2年生存率分别为27.28%和27.93%；PPS集1年生存率分别为61.31%和65.77%，2年生存率分别为29.45%和27.36%。两组患者的1年生存率及2年生存率无统计学差异。此外，本实验还对PP组和GP组非鳞癌患者进行生存分析，结果发现非鳞癌患者FAS集1年生存率分别为64.26%和63.93%，2年生存率分别为28.99%和28.32%；PPS集1年生存率分别为66.07%和63.67%，2年生存率分别为31.46%和27.91%，两组无统计学差异。PP组和GP组非鳞癌患者FAS集中位生存时间分别为509天和508天，即分别为17.0个月和16.9个月（按照每个月30天计算），PPS集分析PP组和GP组中位生存时间分别为523天和511天，即分别为17.4个月和17.0个月，两组亦无统计学差异。就是说我们的研究并没把非鳞癌患者中有效率的优势转化成总生存的优势，可能和病例数少有关系。另外，国外的一些报道称：PP与GP两种方法治疗非鳞癌患者的疗效无明显差异，总生存有明显性差异。在本次研究中，非鳞癌患者两组疗效有明显性差异，而总生存无明显性差异。与国外报道不完全相符，亦可能因为本次研究中腺癌患者约占总患者人数的70%，在二线治疗中两组使用靶向治疗的比例分别为32.53%和35.36%。这对患者的生存时间产生了影响，在一定程度上缩小了两组间的差别。王伟等^[12]^在进行吉西他滨和培美曲塞的比较研究中，鳞癌患者的生存期在吉西他滨组中优于培美曲塞组，而在本组研究中，鳞癌患者样本例数较少，不能直观说明两种治疗方案在鳞癌患者间的疗效比较。综上，从药物疗效分析来看，PP组和GP组疗效相当。

本项临床研究自2008年3月开始至2009年10月所有患者用药结束。其中对照药吉西他滨的用量低于说明书中用量和国外研究中的用量，其用药剂量的确定主要依据为：2008年和2009年《NCCN非小细胞肺癌临床实践指南（中国版）》^[13, 14]^。其中均标明吉西他滨与顺铂联合用药时使用剂量为1, 000 mg/m^2^，d1、d8使用，3周为一化疗周期。而国外（加拿大，美国）有的相关临床研究中对照组吉西他滨联用顺铂的用法用量为：吉西他滨1, 250 mg/m^2^，d1、d8，顺铂75 mg/m^2^，d1，3周为1个周期。但是也有一些为了减轻不良反应，用量稍微保守，即吉西他滨1, 000 mg/m^2^，d1、d8，顺铂75 mg/m^2^，d1，这样对于Ⅲ期-Ⅳ期的患者来说，副反应较少^[15]^。鉴于西方人与国人体质的差异，尤其是出于安全方面的考虑，本临床研究吉西他滨的使用剂量定为1, 000 mg/m^2^。

本临床研究安全性分析结果表明：PP组血小板降低、血红蛋白降低、白细胞减少、脱发、便秘、粒细胞减少、心电图异常、食欲不振、乏力等不良反应的发生率低于GP组。其中血小板降低（*P* < 0.000, 1）、血红蛋白降低（*P*=0.007, 1）、白细胞减少（*P*=0.006, 9）和脱发（*P*=0.011, 5）发生率有统计学意义；重度不良反应（NCI 3-4）PP组白细胞减少发生率10.16%（*P*=0.003, 7）、血小板降低发生率3.91%（*P*=0.018）均明显低于GP组。因GP组以上不良反应发生率高于PP组，所以GP组患者因为不良反应中止试验的患者相对较多，因此用药周期少于试验组。PP组和GP组药物暴露周期的比较分析与安全性分析结果相吻合。该安全性分析结果表明，吉西他滨1, 000 mg/m^2^导致的不良反应较高，并且已经影响受试者用药周期，若采用更高的1, 250 mg/m^2^剂量，将可能给受试者将带来更为严重的不良反应，其利益将受到更大的损害。

本研究与国外Ⅲ期临床研究中3级、4级不良反应比较情况如下：本研究与国外Ⅲ期试验东亚人种亚组相比，血液学毒性发生率高于同期国外研究，国外临床中不良反应如中性粒细胞减少，白细胞减少，血小板减少，贫血等症状的发生率一般在20%以下，而本研究中上述症状的发生率有的在20%以上；而在非血液学毒性中，脱发和全身乏力的症状发生率国外Ⅲ期试验东亚人种亚组基本为10%-20%之间，而本研究脱发和乏力的症状发生率均在10%以下^[15, 16]^。可能是由于实验的患者机体差别较大，对同样的药物所产生的副反应和耐受性均有一定的差异所致。

本研究中心较多，而且各中心入组病例数相差较大（最少为5例，最多为30例）。在方案设计时，考虑到因为中心较多和各中心入组不均衡可能带来的影响，本研究采用了中央随机化分配病例的模式，即把所有中心作为一个中心进行病例分配，因此各中心之间虽然脱落率有一定的差异，但因为采用了中央随机化处理方式，因此对总体疗效和安全性之间的影响在随机化的时候就已消除。

本研究总脱落率为13.39%，其中PP组为13.28%，GP组为13.49%，总体脱落率和两组脱落率均小于20%。因此虽然各中心之间脱落率有一定的波动，但对整体疗效和安全性评价并没有产生实质性影响。另外，本研究中脱落率较高的中心其PP组和GP组的脱落率基本一致，所以即使有影响，最终对试验组和对照组之间的差值也不会产生影响，因此本研究的科学性和严谨性不会受到脱落率的影响。

结合本研究的结果进行分析，提示培美曲塞联合顺铂方案一线治疗晚期NSCLC对比吉西他滨联合顺铂方案在疗效方面相似，但是可减少一些毒副反应如血小板减少、血红蛋白含量降低、白细胞减少、脱发等。所以由于较好的疗效和较轻的不良反应，值得临床推广。
